# Metabolic Syndrome among Patients with Psoriasis Attending the Outpatient Department of Dermatology, Venereology and Leprology in a Tertiary Care Centre: A Descriptive Cross-sectional Study

**DOI:** 10.31729/jnma.8225

**Published:** 2023-07-30

**Authors:** Pratichya Thapa, Sangeeta Paudel, Shraddha Uprety, Santosh Timalsina

**Affiliations:** 1 Department of Dermatology, Venereology and Leprology, Chitwan Medical College and Teaching Hospital, Bharatpur, Chitwan, Nepal; 2 Department of Biochemistry, Chitwan Medical College and Teaching Hospital, Bharatpur, Chitwan, Nepal

**Keywords:** *inflammation*, *metabolic syndrome*, *psoriasis*

## Abstract

**Introduction:**

Metabolic syndrome, a constellation of features such as obesity, hypertension, dyslipidemia and insulin resistance is of common occurrence in patients with psoriasis. Systemic inflammation is supposed to play a significant role in both conditions. The co-occurrence of metabolic syndrome and psoriasis can result in clinical consequences. The aim of this study was to find out the prevalence of metabolic syndrome among patients with psoriasis attending the Outpatient Department of Dermatology, Venereology and Leprology in a tertiary care centre.

**Method:**

A descriptive cross-sectional study was conducted in the Outpatient Department of Dermatology, Venereology and Leprology from 4 July 2022 to 4 May 2023. The ethical approval was obtained from Institutional Review Committee (Reference number: CMC-IRC/078/079-300). Patients aged more than 18 years diagnosed with psoriasis who provided written consent were enrolled in the study. Pregnant female patients and those who did not give informed consent were excluded from the study. Convenience sampling method was used. Point estimate and 90% Confidence Interval were calculated.

**Results:**

Among 72 patients with psoriasis, the prevalence of metabolic syndrome was 15 (20.83%) (12.98-28.68, 90% Confidence Interval). Obesity 13 (86.67%) and hypertriglyceridemia 13 (86.67%) were the frequent most individual components of metabolic syndrome.

**Conclusions:**

The prevalence of metabolic syndrome among psoriatic patients was lower than other studies done in similar settings.

## INTRODUCTION

Metabolic syndrome (MetS) constitutes a cluster of hypertension, central obesity, insulin resistance, and atherogenic dyslipidemia.^1^ The prevalence of MetS in psoriatic patients is quite significant, with reports ranging from 20-50%.^2,3^ Systemic inflammation plays a significant role in the causation of both these conditions.

Studies on the prevalence of MetS and psoriasis from Nepal are few and far between. Thus, awareness of primary preventive aspects of MetS components could reduce the systemic conditions that can arise later in these patients.

The aim of this study was to find out the prevalence of metabolic syndrome among patients with psoriasis attending the Outpatient Department of Dermatology, Venereology and Leprology in a tertiary care centre.

## METHODS

A descriptive cross-sectional study was conducted from 4 July 2022 to 4 May 2023 in the Outpatient Department of Dermatology, Venereology and Leprology at Chitwan Medical College and Teaching Hospital, Bharatpur, Chitwan, Nepal. The ethical approval was taken from the Institutional Review Committee (Reference number: CMC-IRC/078/079-300). Patients aged >18 years diagnosed with psoriasis who provided written consent were enrolled in the study. Pregnant female patients and those who did not give informed consent were excluded from the study. Convenience sampling method was done. The sample size was calculated using the following formula:


$$\begin{array}{ll} \text{n} & ={\text{Z}}^{2}\times \frac{\text{p}\times \text{q}}{{\text{e}}^{\text{2}}}\\ & =1.64^{2}\times \frac{0.5\times 0.5}{0.1^{2}}\\ & =67 \end{array}$$

Where,

n= minimum required sample size
Z = 1.64 at 90% Confidence Interval (CI)
p = prevalence taken as 50% for maximum sample size calculation
q = 1-p
e = margin of error, 10%

The calculated sample size was 67, However, 72 patients were included in this study.

The sociodemographic and clinical characteristics of patients were noted. Clinical measurements such as height, weight, blood pressure and waist circumference were recorded, following standard guidelines. The psoriatic area and severity index (PASI) scoring was calculated to assess the severity of psoriasis after taking into account the surface area involved and the character of the lesions (erythema, induration and scaling).^4^

The patients were sent for estimation of fasting blood sugar and lipid profile parameters the following day. The patients were diagnosed with metabolic syndrome if the NCEP ATP III criteria were met, that is-three or more of the following five parameters: waist circumference over 40 inches (men) or 35 inches (women), blood pressure over 130/85 mmHg or under treatment, fasting triglyceride (TG) level over 150 mg/dl or under treatment, fasting high-density lipoprotein (HDL) cholesterol level less than 40 mg/dl (men) or 50 mg/dl (women) and fasting blood sugar over 100 mg/dl or under treatment.^5^

Data were entered and analysed using IBM SPSS Statistics version 20.0. Point estimate and 90% CI were calculated.

## RESULTS

**Figure 1:**
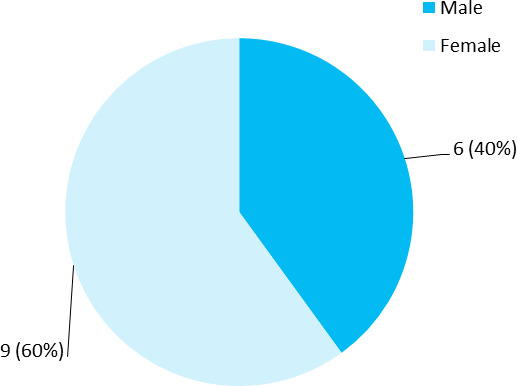
Distribution according to gender (n = 15).

Among 72 patients with psoriasis, the prevalence of metabolic syndrome was 15 (20.83%) (12.98-28.68, 90% CI). The mean age was 49.51+13.65 years with a range of 20-78 years. Out of 15, 6 (40%) were male (Figure 1).

Among 15 patients, the commonest age group was 4160 years 12 (80%). Hypertension was the commonest pre-existing comorbidity 5 (33.33%) (Table 1).

**Table 1:** Different characteristics of the patients (n = 15).

Variables	n (%)
**Age (years)**	
20-40	-
40-60	12 (80)
61 and above	3 (20)
**Comorbidities**	
Hypertension (HTN)	5 (33.33)
Type 2 diabetes mellitus (T2DM)	4 (26.67)
Hypothyroidism	2 (13.33)
**Personal history**	
Current smoker	4 (26.67)
Current alcohol consumer	3 (20)

The prevalence of MetS was higher among patients with mild psoriasis (Table 2).

**Table 2:** Severity grading based on PASI (n= 15).

Variables	n (%)
Mild psoriasis	13 (86.67)
Moderate psoriasis	1 (6.67)
Severe psoriasis	1 (6.67)

The most common MetS components among the psoriatic patients were elevated triglyceride 13 (86.67%) and high waist circumference 13 (86.67%) (Table 3).

**Table 3:** Distribution of individual MetS components (n= 15).

Variables	n (%)
Elevated triglyceride	13 (86.67)
High waist circumference	13 (86.67
Hypertension	10 (66.67)
Abnormal fasting blood glucose	8 (53.33)
Low HDL-c	3 (20.00)

## DISCUSSION

The prevalence of MetS among psoriatic patients in our study was 15 (20.83%) which was lower than in studies done in similar settings. This finding was comparable to various studies that have reported the prevalence in the range from 28-40%.^3,6-8^ MetS among psoriatic patients is rapidly rising globally which has important clinical implications. Psoriasis, being a chronic inflammatory disease is characterized by various systemic inflammatory changes which may in turn contribute to atherogenesis and insulin resistance in these patients. Various genetic studies have shown that psoriasis and cardiovascular diseases (CVDs) have a common involvement of various inflammatory mediators like TNF-alpha and IL-1.^9^

The mean age of our study population was 49.51±13.65 years with a range of 20 to 78 years. This was similar to a study done in the mid-west part of Nepal.^10^ The mean age of presentation reflected similarity in central Nepal and mid-west Nepal. A majority of our patients were females. Female predominance among psoriatic patients as observed in our study was reported in a study from Nepal.^10^ However, few of the studies have described male predominance.^11,12^ Females are more anxious when it comes to aesthetics and looks. This could be the reason for more number of female patients in our study.

The prevalence of MetS was found to be the highest in the mild psoriasis group 13 (86.67%), however, the sample size was very small to reinforce this finding. One of the studies has reported no correlation between the severity of psoriasis and the prevalence of MetS,^8^ suggesting that MetS can exist despite the severity of the disease. In another study, patients with more severe diseases had a greater risk of MetS.^13^ It implicates the need for routine testing for MetS in all patients with psoriasis. Consistent with an international study,^8^ our study revealed elevated triglyceride levels and high waist circumference as the most common MetS components. Another study also demonstrated the risk of hypertriglyceridemia to be higher in psoriatic patients.^14^ This gives us an indication that every psoriatic patient should be assessed for MetS, along with the individual MetS components, because many of them are amenable to pharmacological and lifestyle management.

In our study, obesity (defined based on waist circumference) has been found to be more common in female patients compared to male patients. Obesity and psoriasis can go hand in hand as one increases the incidence and severity of the other. Obesity constitutes of overproduction of inflammatory cytokines such as tumour necrosis factor-a (TNF-α), and interleukin (IL)- 1,6 and 8 in adipocytes, which is directly related to the pathogenesis of psoriasis.^15^

It is noteworthy that individual MetS components could be independently existent in psoriatic patients, and therefore they should be assessed in every psoriatic patient. Awareness of the primary preventive aspects of MetS such as early baseline investigations and subsequent pharmacological treatment, healthy diet plans and regular physical activity could reduce the disease burden in these patients.

Our study was limited to a small number of patients coming to our centre. A larger population would have been more representative. Furthermore, a comparative study with the general population could be done to evaluate the scenario of metabolic syndrome in psoriatic patients versus non-psoriatic patients.

## CONCLUSIONS

The prevalence of metabolic syndrome among patients with psoriasis was lower than other studies done in similar settings. Thus, a practice of screening all psoriatic patients for metabolic syndrome and timely intervention can decrease the health burden associated with psoriasis.
